# Case of asteroid hyalosis that developed severely reduced vision after cataract surgery

**DOI:** 10.1186/s12886-017-0467-6

**Published:** 2017-05-15

**Authors:** Ryosuke Ochi, Bumpei Sato, Seita Morishita, Yukihiro Imagawa, Masashi Mimura, Masanori Fukumoto, Takaki Sato, Takatoshi Kobayashi, Teruyo Kida, Tsunehiko Ikeda

**Affiliations:** 1 Department of Ophthalmology, Osaka Kaisei Hospital, Osaka-City, Osaka Japan; 2 Department of Ophthalmology, Osaka Medical College, 2-7 Daigaku-machi, Takatsuki City, Osaka 569-8686 Japan

**Keywords:** Asteroid hyalosis, Cataract surgery, Vitreous surgery, Posterior vitreous detachment

## Abstract

**Background:**

To report our findings in a patient with asteroid hyalosis (AH) who had a severe reduction of his visual acuity following cataract surgery. The vision was improved by vitreous surgery.

**Case presentation:**

The patient was an 81-year-old man. Following cataract surgery on his left eye, his decimal best-corrected visual acuity (BCVA) was markedly reduced from 0.2 to 0.02. A large number of asteroid bodies (ABs) was observed to be concentrated on the posterior surface of the implanted intraocular lens. Ultrasound B-mode images showed turbidity of the vitreous that was denser in the anterior vitreous where the ABs were concentrated. During vitrectomy, the ABs were observed to be concentrated in the anterior vitreous cavity, and a complete posterior vitreous detachment (PVD) was present. After vitrectomy successfully removed the ABs, the visibility of the fundus improved and the BCVA recovered to 1.0.

**Conclusion:**

We suggest that the visual impairment after the cataract surgery was due to the concentrated ABs in the anterior vitreous cavity. The clustering of the ABs in the anterior vitreous cavity was most likely caused by the PVD that developed during the cataract surgery.

## Background

Asteroid hyalosis (AH) is a degenerative vitreous disease that is common among the elderly and was first described by Benson in 1894 [1]. It is characterized by a mild liquefaction of the vitreous body and a reduced likelihood of a posterior vitreous detachment (PVD) [2, 3]. Ophthalmoscopic examinations of eyes with AH show many light-yellow plaques which give the appearance of stars or asteroid bodies (ABs) shining in the night sky. However, the ocular asteroids can be mobile. The presence of the ABs in the vitreous cavity can reduce the visibility of the fundus although they rarely cause vision reduction and myodesopsia [4]. Thus, it is rare for this disease to be treated with vitreous surgery.

We report our findings in a patient with AH whose best-corrected visual acuity (BCVA) decreased severely after cataract surgery but then markedly improved after vitreous surgery.

## Case presentation

### Patient

An 81-year-old man.

### Chief complaint

Decreased vision in the left eye.

### History of present illness

The patient underwent phacoemulsification and intraocular lens (Alcon Acosov UV posterior chamber lens; model number, MA 60) implantation for a cataract in his left eye at another hospital on August 26, 2014. Although the surgery was completed without complications, his decimal best-corrected visual acuity (BCVA) decreased from 0.2 to 0.02 following the surgery. He was then referred to the Department of Ophthalmology at Osaka Medical College Hospital, Takatsuki, Japan on October 15, 2014.

### Past medical history

The patient was not diabetic. He did not report experiencing floaters.

### Family history

No significant family history.

### Findings on initial ocular examination

**Fig. 1:**
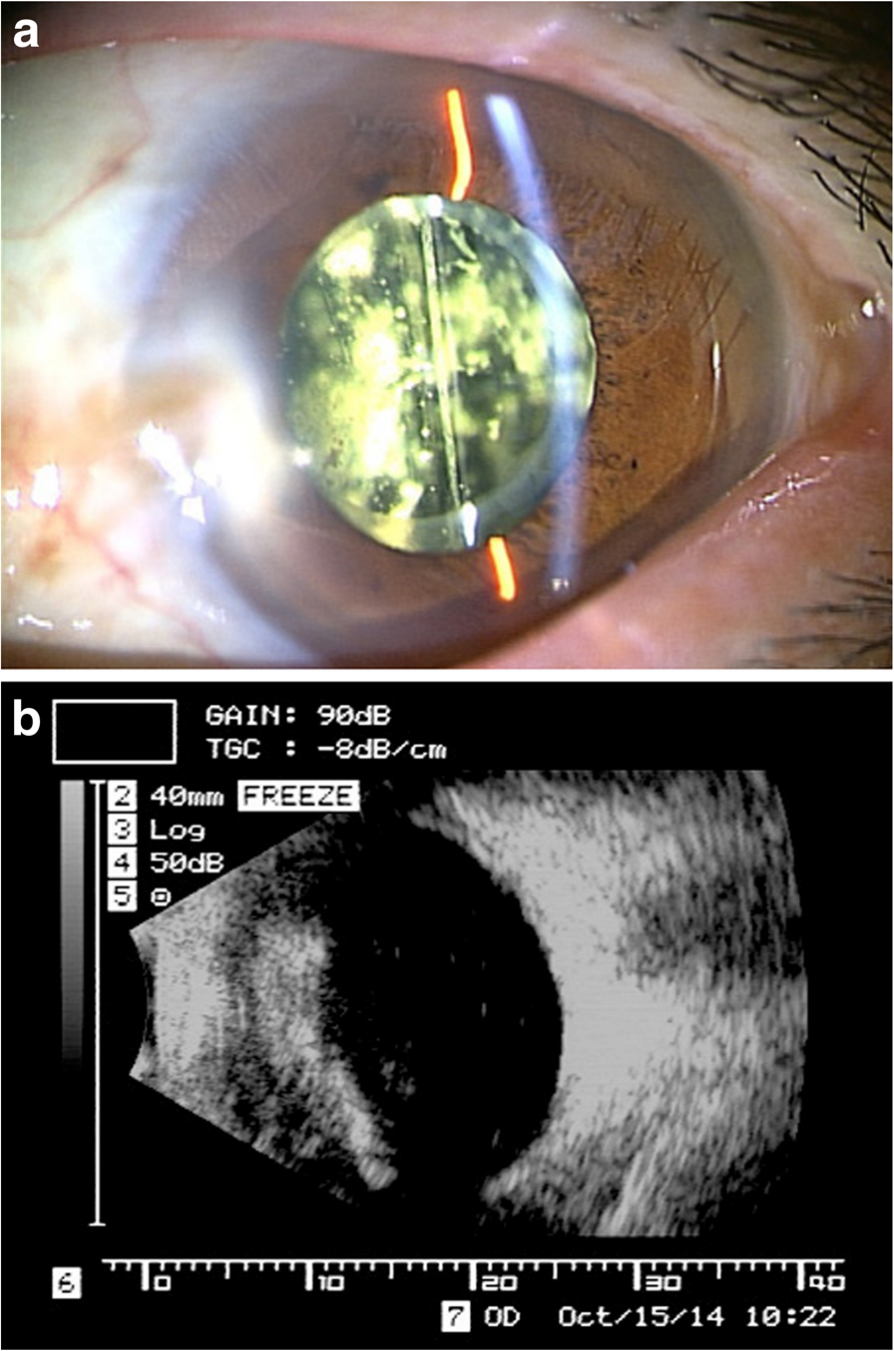
Ocular findings in a patient with asteroid hyalosis (AH) after successful cataract surgery. The asteroid bodies (ABs) appear to be concentrated in the anterior vitreous cavity of the left eye (**a**), and an ultrasonic B-mode image shows a shadow that appears to be a cluster of asteroid bodies (ABs) in the anterior vitreous cavity (**b**)

His initial examination of the left eye showed that his decimal BCVA was 0.02, the intraocular pressure was 14.0 mmHg, and the intraocular lens was well centered. A dense concentration of ABs was observed in the anterior vitreous cavity of the left eye, and the visibility of the fundus was very poor ophthalmoscopically (Fig. 1a). An ultrasound B-mode examination showed a shadow that appeared to be a cluster of ABs concentrated in the anterior vitreous cavity (Fig. 1b). Spectrum domain optical coherence tomography (SD-OCT) was attempted but the resulting images were blurred and indistinct.

### Follow-up course

**Fig. 2:**
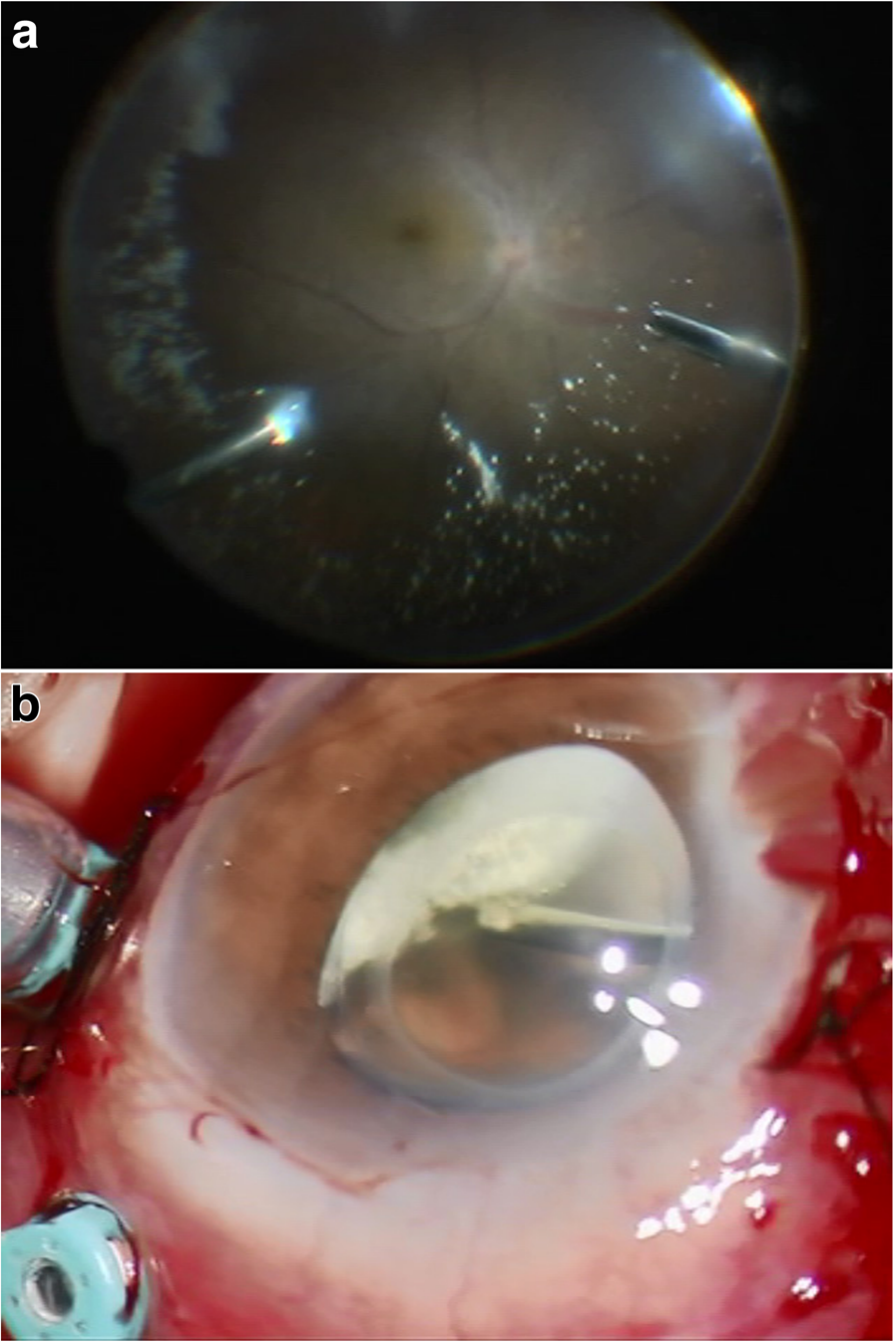
Intraoperative findings. The ABs are concentrated in the anterior vitreous cavity and a complete posterior vitreous detachment is present (**a**, **b**)

On November 4, 2014, the patient underwent vitrectomy on his left eye using a 25-guage system. During the vitrectomy, a complete PVD was detected to be already present, and the ABs were found to be concentrated in the anterior vitreous (Fig. 2a). After injecting triamcinolone acetonide, it was noted that no vitreous gel remained on the retinal surface but cystoid macular edema (CME) was present. The vitreous body including the ABs just posterior to the intraocular lens were excised, and the peripheral vitreous and ABs were removed with compression on the sclera under microscopic coaxial illumination (Fig. 2b).

One day after the vitrectomy, the vision was markedly improved, and at 4-days the BCVA had improved to 0.7. The CME that was detected intraoperatively was still present although the degree of edema was reduced. The CME gradually disappeared, and at 5-months after the vitrectomy, the BCVA had improved to 1.0.

## Discussion

In general, the ABs have little effect on visual function, and it is rare to treat AH with vitreous surgery. The reason why the subjective symptoms such as decreased vision or floaters rarely occur in eyes with AH is that the density of ABs in the vitreous cavity is relatively low [4]. In addition, the ABs are present only in the vitreous gel and are not present in the liquid vitreous, Cloquet’s canal, or posterior to a vitreous detachment [2]. In our case, it is unclear whether the PVD existed before the cataract surgery, but it is more likely that the cataract surgery caused the PVD [5]. We suggest that the visual impairment developed because the PVD forced the residual vitreous to move to the anterior vitreous cavity. This resulted in concentrating the ABs in the anterior vitreous cavity closer to the nodal point of the eye.

Even when the fundus visibility is poor in eyes with AH, it is common for the fluorescein angiographic and OCT images to be clear [6, 7]. In particular, OCT is excellent at detecting macular diseases such as age-related macular degeneration, epiretinal membrane, macular hole, and CME in eyes with AH which are all difficult to detect ophthalmoscopically. Moreover, OCT is quite useful for detecting the changes that cause the vision reduction in AH patients.

In our patient, clear SD-OCT images of the retina could not be obtained prior to the vitreous surgery. We suggest that this was because the ABs were so concentrated in the anterior vitreous cavity near the nodal point that not enough light could pass through the ABs to form a sharp tomographic image. Although the CME may have also caused some of the decreased VA in this eye, the vision did improve significantly despite the presence of the CME after the surgery. Thus, the concentrated ABs in the anterior vitreous were the more likely cause of the reduced vision. However, the true extent of the vision being affected due to asteroid hyalosis may be difficult to explain due to the inability to image the macula before the vitrectomy.

There have been several reports of AH cases in which the BCVA improved after vitreous surgery [8–10]. However, only a few of these studies reported on the exact cause of the reduced vision prior to the surgery. Jingami et al. reported on a AH patient with retinitis pigmentosa who had a decrease in the VA after cataract surgery [11]. Just as in our case, there was a significant improvement of the VA after vitreous surgery. They suggested that the cause of the decreased VA after the cataract surgery was that changes in the AB distribution caused by a change in the shape of the vitreous body due to the cataract surgery. However, it was reported that the posterior vitreous body was not detached when it was examined during vitreous surgery [12, 13]. Reviewing the published cases in which vision was improved by vitreous surgery, we were unable to find publications where the posterior vitreous was clearly detached as it was in our case.

The posterior vitreous is usually not detached in eyes with AH, and in cases of AH accompanied by advanced diabetic retinopathy in particular, there are many cases of extremely strong vitreoretinal adhesions and surgery is known to be very difficult [13]. Mochizuki et al. found that even in AH cases where it initially appeared that a PVD was present, it was common for the vitreous cortex to be found in the posterior retina and for vitreoschisis to be present [3]. However, even in AH cases, there are cases such as our case in which a complete PVD had occurred and the ABs were concentrated in the anterior vitreous. Thus, we suggest that in such cases vitreous surgery should be performed especially if the VA is reduced.

## Conclusion

We suggest that the visual impairment after the cataract surgery was due to the concentrated ABs in the anterior vitreous cavity and vitreous surgery should be performed especially in such cases.

## Abbreviations

ABs: Asteroid bodies
AH: Asteroid hyalosis
BCVA: Best-corrected visual acuity
CME: Cystoid macular edema
PVD: Posterior vitreous detachment
SD-OCT: Spectrum domain optical coherence tomography
